# The prevalence of obstructive sleep apnoea risk factors in individuals with cognitive impairment: south London cross-sectional study

**DOI:** 10.1192/bjb.2022.38

**Published:** 2023-08

**Authors:** Leiah Kirsh, Rebecca Cox, Heloise Mongue-Din, Robert Lawrence

**Affiliations:** 1 St. George's, University of London, UK; 2 South West London and St George's Mental Health NHS Trust, Research & Development, UK

**Keywords:** Dementia, sleep disorders, clinical governance, comorbidity, cognitive neuroscience

## Abstract

**Aims and Method:**

We aimed to determine the prevalence of risk factors for obstructive sleep apnoea (OSA) in patients with mild cognitive impairment (MCI) or dementia. Using patient records across a 5 year period, we identified 16 855 patients with dementia or MCI. We gave scores for keywords relating to each modified STOP BANG parameter in patient progress notes. Based on individual scores, we stratified the population into groups with low, intermediate and high risk of OSA.

**Results:**

Our population had a high prevalence of risk factors and consequently high risk scores for OSA (18.21% high risk). Parameters directly related to sleep had a low prevalence.

**Clinical implications:**

The risk of developing or having OSA is high among patients with MCI and dementia. Low sleep parameter frequency probably suggests poor documentation of sleep rather than true prevalence. Our findings support the implementation of the STOP BANG or a similar screening tool as a standardised method to identify OSA risk in memory clinics.

Obstructive sleep apnoea (OSA) is a sleep disorder characterised by episodes of partial or complete airway blockage while sleeping.^1^ Among older patients, the prevalence of OSA is about 50% in males and 25% in females,^2^ and over 60% of patients with mild cognitive impairment (MCI) or dementia are thought to have sleep disorders.^1,3^ This is particularly worrying because those with sleep disturbances have been found to have a 75% increased risk of developing dementia.^4^ Unfortunately, patients with cognitive impairment are not routinely screened for OSA; thus, a large proportion of people with OSA are undiagnosed and untreated.^5^ Some studies have cited poor sleep quality as an association with MCI and dementia, rather than sleep-disordered breathing.^6^

A commonly used OSA screening tool is STOP BANG, which gives patients a score for each of the specified risk factors and clinical signs. It remains unclear how many at-risk patients would benefit from the implementation of STOP BANG to identify OSA risk factors in memory services. The specific factors responsible for the increased OSA risk seen in patients with MCI or dementia have not been detailed. Understanding which risk factors are of interest in a clinical setting could facilitate targeted interventions to improve cognitive impairment symptoms. Consequently, our study set out to measure the prevalence of a range of OSA risk factors in a sample population with a diagnosis of MCI or dementia.

## Method

### Study design

This was a retrospective cross-sectional study. This study was approved by the Joint Research and Enterprise Services at St George's, University of London (ref: SE0005). This study was performed in accordance with the Declaration of Helsinki. The project was exempt from ethics approval as the research was limited to secondary use of de-identified information previously collected in the course of normal care, without an intention to use it for research at the time of collection. For the same reason, adult participant consent was not required.

### Population

The inclusion criteria for our population consisted of out-patients and in-patients with a diagnosis of dementia (F03) or MCI (F067) who had accessed South London and St George's Mental Health NHS Trust services between 1 January 2013 and 31 December 2017. This produced a total of 16 855 patients that were included in the study.

### Measure

A modified STOP-BANG sleep apnoea assessment tool was used to examine the prevalence of OSA risk factors in our population. STOP BANG is an eight-item risk stratification tool relating to the clinical features and risk factors of OSA. Individual scores range from 0 to 8, with a single point given for each positive finding of a parameter. This particular screening tool was selected because of its high predictive probability of 0.77 (95% CI: 0.69–0.84) with a STOP BANG score of 5.^7^ Parameters include snoring, tiredness, observed apnoea, high blood pressure, body mass index (BMI), age, neck circumference and male gender. We included additional risk factor parameters (diabetes,^8^ a past cardiovascular event,^9^ and being female and aged over 55 years^10^) to form a modified STOP BANG with a greater sensitivity for quantifying OSA risk. These risk factors were selected as expansions of the existing variables of BMI, high blood pressure and age, respectively. The score ranges for risk were kept the same as those of the original STOP BANG.

### Procedure

Participants were isolated using the Clinical Record Interactive Search (CRIS) system. CRIS is a research platform gathering anonymised electronic health records from 12 National Health Service mental health trusts across the UK.^11^ Data from an electronic patient record software, RiO, were transferred to CRIS in an anonymised fashion. Data were limited to notes within the mental health trust secondary care services and did not include physical health or general practice records.

SQL (structured query language; a programming language) was used to interrogate the CRIS database and isolate the sample population that met our inclusion criteria. Once the sample had been isolated, the text in the progress notes was analysed to locate mentions of keywords relating to the modified STOP BANG parameters, as detailed in Table 2.

Age at the most recent visit (within the specified time frame) and gender were retrieved from patients’ demographic details. Ethnicity data were also collected from the patient demographic details. Neck circumference was omitted, as this is not routinely measured in memory services. Keywords were used to include the various ways healthcare professionals may reference the risk factors in patient notes for the following parameters of the modified STOP BANG: snoring, tiredness, observed to stop breathing while sleeping, pressure (hypertension), BMI (obese), diabetes, cardiovascular events (Table 2). Keywords with the Boolean operator ‘*’ were used where different endings of the word might be written. Many of the parameters of interest are not routinely recorded or recorded in a uniform manner; therefore, the keywords were selected in an attempt to anticipate various spellings, synonyms and phrases. For example, when looking at cardiovascular events, we used the search term ‘myocardial inf*’ to account for common spelling errors of the word infarction.

Excessive alcohol consumption (EAC) and smoking were independently measured. These variables were not used to calculate risk scores. The presence of EAC was determined if the key phrases ‘excessive alcohol,’ ‘heavy alcohol,’ ‘alcohol misuse’ or ‘history of alcohol’ or the ICD-10 diagnosis code F10 (alcohol-related disorders) were identified in the progress notes. Smoking was determined by the key phrases ‘heavy smoking,’ ‘smokes heavily’ or ‘heavy smoker’.

This information was exported for further descriptive analysis of our sample and of the prevalence of OSA risk factors.

### Analysis

Our primary outcome was the prevalence of each modified STOP BANG parameter or OSA risk factor. We then grouped individuals into low, intermediate or high OSA risk groups based on their scores on the modified STOP BANG (low risk, scores of 0–2; intermediate risk, scores of 3–4; high risk, scores of 5+).

### Missing data

Missing data for the modified STOP BANG parameters were assumed to be a negative finding for the given risk factor, as this represented no documentation or the use of alternative words not included in the keyword search. We tried to overcome this by including several keywords as described above in the Measure subsection.

## Results

The inclusion criteria identified a sample of 16 855 patients (38% male and 62% female). For both male and female patients, the greatest percentage of individuals were in the intermediate risk group (Table 1); however, a greater percentage of males were high risk than females (20.6% of males, 16.8% of females) (Table 1). A large majority (84%) of the sample population was of White ethnicity (Table 1). White patients had a greater prevalence of intermediate risk (79%) than Asian or Black patients (71% and 67%, respectively). Seventeen per cent of White patients, 27% of Asian patients and 29% of Black patients were high risk.

**Table 1:** Characteristics of risk groups

		OSA risk group (score)		
	Total	Low (0–2)	Intermediate (3–4)	High (5+)
No. (%)	16855 (100)	566 (3.36)	13219 (78.43)	3070 (18.21)
Age, mean, years	83	79	83	82
Male sex, *n* (% of males); 38%	6400 (100.00)	210 (3.28)	4908 (76.69)	1333 (20.83)
Female sex, *n* (% of females); 62%	10455 (100.00)	356 (3.41)	8311 (79.49)	1737 (16.61)
Heavy smoking, %		0.71%	1.69	3.32
Excessive alcohol consumption, %		5.48	4.63	8.31
Ethnicity: *n* (% of total ethnicity)				
White, 84%	14080 (100.00)	480 (3.40)	11182 (79.40)	2418 (17.20)
Asian, 6%	1003 (100.00)	25 (2.49)	709 (70.69)	269 (26.82)
Black, 4%	739 (100.00)	28 (3.79)	493 (66. 71)	218 (29.50)
Other, 4%	669 (100.00)	14 (2.09)	519 (77.58)	136 (20.33)
Unknown or not stated, 2%	364 (100.00)	19 (5.22)	316 (86.81)	136 (37.36)

The high-risk group also had higher prevalence of EAC (8.31%) and smoking (3.32%) than the low-risk group (5.48% and 0.7%, respectively), although the overall prevalence of EAC and smoking in the sample was low (Table 1). Furthermore, the low-risk group had a higher prevalence of EAC (5.48%) than the intermediate-risk group (4.63%). The prevalence of comorbidities detailed in the modified STOP BANG was highest in the high-risk group. In this group, 60.2% had diabetes, 85.2% had hypertension and 100% had cardiovascular comorbidity (as determined by the presence of a cardiovascular event) (Table 1).

The three most prevalent risk factors were age >50 (99%), cardiovascular events (97%), and female gender plus being >55 years old (61%) (Table 2). Data for neck circumference were not obtained. The least frequently present risk factors were snoring (0.9%), having been observed to stop breathing while sleeping (0.3%) and obesity (2.8%). Risk scores were calculated for individual patients based on their total number of risk factors: 3070 (18.2%) were high risk, 13 219 (78.4%) were intermediate risk and 566 (3.36%) were low risk (Table 1).

**Table 2:** Risk factors

Risk factor	Keywords searched	Frequency (%)
Snoring	Snoring, snore	157 (0.93)
Tiredness	Is tired, was tired, fatigue, lack of sleep, drowsy, sleepy, sleep deprived, poor sleep	4102 (24.34)
Observed to stop breathing while sleeping	Stopped breathing, CPAP, sleep apnea, apnea	56 (0.33)
Pressure (hypertension)	High BP, high blood pressure, hyperten*	5028 (29.83)
BMI (obese)	Overweight, obese, high BMI, BMI >35, BMI >26	477 (2.83)
Age >50 years	N/A	16740 (99.32)
Neck circumference (>40 cm)	N/A	N/A
Gender (male)	N/A	6451 (38.27)
+Diabetes	Diabete*, diab*	2559 (15.18)
+Cardiovascular event	MI, myocardial infarct*, myocardial infract*, myocardial inf*, heart attack, stroke, heart problem, vascular accident, vascular problem, CVA, cardiac problem, TIA, ischemia, drop attack, cardiovasc*, cardiac arrest, heart failure	16 355 (97.03)
+Female >55 years old	N/A	10 331 (61.29)

## Discussion

### Interpretation of findings

Our results suggest that there is a high prevalence of OSA risk factors among patients with dementia and MCI, which supports previous research indicating a higher prevalence of OSA in this population.^1–4,12^ Although we were unable to compare these patients with the general population, these findings highlight that this group would benefit from routine OSA screening. At present, patients may be asked about their sleep at their initial consultation; however, this is not consistently carried out and recorded. The low frequency of the variables ‘snoring’ (0.9%) and ‘observed to stop breathing’ (0.3%) represent poor sleep history documentation for patients with memory impairment. This is in accordance with the results of an Alzheimer's Disease Neuroimaging Initiative study that measured sleep-disordered breathing using self-reporting (clinician documentation).^2^ This cohort was found to have a prevalence of 7%, well below the general prevalence in older adults (50% in males and 25% in females), also highlighting an underreporting of sleep disturbances in this population.

Furthermore, many of the parameters, including neck circumference, blood pressure and BMI, are not routinely accounted for in memory services. As such, risk scores are likely to be higher than reported, as these factors are not considered in the scoring. It is possible that many high-risk patients are not being identified or referred for further sleep assessment. With routine use of tools such as the modified STOP BANG, patients will be more likely to receive the indicated treatment or investigations for OSA.

Of note, among Black, Asian and minority ethnic (BAME) individuals, there was a greater percentage of individuals in the high-risk group for OSA. However, the BAME population was severely underrepresented in this sample. It is likely that the results were confounded by selection bias due to the distinct characteristics of the individuals that did attend memory services. For example, it is possible that this group had more severe cognitive impairment than those that did not attend.

Those in the high-risk group had the highest prevalence of heavy smoking, which would support previous findings of this being a risk factor for OSA.^13^ The seemingly random distribution of the prevalence of EAC suggests that the findings regarding this factor may have been due to chance. Overall, the low prevalence of both factors in our sample suggests that these risk factors may not be as relevant as others in understanding the rates of OSA in MCI or dementia. It should be noted that the search terms used in our study for heavy smoking were limited. The term ‘heavy smoking’ is subjective; therefore, the definition may vary among healthcare professionals. This affects the validity of our findings. As heavy smoking and EAC have been previously identified as risk factors for OSA, we suggest that further research is required to outline their relevance in patients with MCI and dementia.

Using the modified STOP-BANG, we were able to identify a large proportion (96.64%) of the sample as being at intermediate to high risk of developing OSA. This is likely to be in accordance with previous studies showing that 60% of patients with dementia or MCI also have OSA. Although the presence of OSA was not determined, this study does highlight that this population has many factors suggestive of OSA. In addition, the use of different sleep disorder screening tools prevents direct comparisons among studies.

A systematic review concluded that patients with MCI do not have more risk factors than the general population, hypothesising that the pathophysiology of MCI may alter the strength of the association of some risk factors.^14^ Another study suggested that although the prevalence of OSA is much higher in patients with MCI, the scores of these patients on sleep disorder subscales did not differ compared with controls.^15^ This may be the reason that other studies have failed to capture the increased risk. Although we did not compare the modified STOP BANG scores with controls, our findings contrasted with those of others as we did observe high scores on our risk-factor-based OSA scale. It may be that the modified STOP BANG contains parameters more appropriate to the measurement of OSA in this population.

The literature has also suggested that poor sleep overall is the true cause of the association with MCI.^6^ This may hold true, but our findings suggest that the poor sleep subtype OSA is likely to be highly prevalent among those with MCI and dementia. This is in line with other findings suggesting that sleep-disordered breathing is the most prevalent form of sleep disturbance in patients with MCI, Alzheimer's dementia, vascular dementia, frontotemporal dementia and Lewy body/Parkinson's disease dementia.^12^

A myriad of risk factors and associations have been proposed as explanations for this link.^1^ However, to our knowledge, there has been limited research looking at several risk factors in the same sample. Our study found that most patients had three or more risk factors. There may be a synergistic relationship between the characteristics present in populations with cognitive impairment; thus, future research should investigate potential clustering patterns of risk factors to better understand their combined effect size.

### Strengths and limitations

One limitation of using retrospective data from the CRIS database is that patient progress notes record clinical observations, which include elements of subjectivity. Variability in patient history collection and recording also made it difficult to confirm the presence of risk factors. Our keyword search attempted to capture commonly used descriptions of various risk factors. It is important to acknowledge that these lists were not exhaustive. In addition, we were looking for the presence of variables that are not routinely collected. Thus, an absence from our keyword search does not necessarily correlate with an absence of the variable. It could be that the variable was not assessed or was documented in a way that our search could not detect. Therefore, we believe a large percentage of patients that scored as intermediate risk were high risk. A positive word search also did not necessarily correlate with a positive finding of the STOP BANG variable. Keyword searches were not proofread for negative findings or context. A positive word search reflected the identification of that word in patient notes. For example, when searching for ‘snoring,’ the sentence could have been ‘does not report snoring’ in the progress notes text. However, given that these parameters are not routinely assessed, we believe that documentation is much more likely to indicate their presence. Therefore, any negative findings would have had minimal impact on our results.

In addition, the study did not use a comparator group. Therefore, we cannot conclude that this population's risk is significantly higher than that of the general population; however, we have found that this population is at high risk. OSA has deleterious effects on those with MCI and dementia, making its identification more urgent in this population compared with the general population. If this condition is untreated, patients with OSA will have increased rates of decline and symptom onset.^2^

### Moving forward

Patients with cognitive impairment exhibit high frequencies of risk factors associated with OSA; however, further longitudinal research is needed to estimate the role of multiple risk factors and risk factor clusters in patients with MCI and dementia. We are not aware of any prospective studies that would help to quantify the extent and significance of specific risk factors in patients with cognitive impairment. Future studies could also explore the strength of the associations of smoking and alcohol use with increased risk in this population. In addition, increased BAME representation in studies could establish the true existence and degree of risk variation across ethnicities. The appropriateness of STOP BANG, and potential additional risk factors, should also be explored for this patient group.

Our findings agree with previous literature that indicate the need for special attention to patient sleep in memory clinics. We suggest that mental health memory clinics, such as those in South West London and St George's Mental Health NHS Trust, implement a modified STOP BANG assessment as part of a standardised cognitive impairment evaluation. The findings highlight the need for routine OSA screening for individuals with MCI and dementia. There is currently no systematic screening for OSA in UK memory services; therefore, this group of patients may not have equal access to referrals and treatment for OSA. Previous research has stated that OSA is commonly undiagnosed and untreated;^5,7^ our findings suggest that this is also true for this population. The delay in diagnosis and treatment for OSA emphasises the need for a practical and reliable method of identifying patients at high risk of OSA. The STOP BANG was specifically designed as a reliable and easy-to-use screening tool to quantify the risk factors predicting OSA.^16^ The STOP BANG has also been widely validated in various populations, which is why we would propose its introduction as a standardised screening tool for use at initial memory assessments.

Screening lends itself to false positives and unnecessary referrals to sleep services. To mitigate this, we recommend the following actions based on risk score: patients with moderate and high risk scores should discuss lifestyle interventions for OSA^17^ with their general practitioner. High-risk patients should be referred to sleep services to determine their eligibility for and tolerance of treatments such as continuous positive airway pressure (CPAP).

## Data Availability

The data that support the findings of this study are available from the corresponding author, Leiah Kirsh, upon reasonable request.

## About the authors

**Leiah Kirsh,** MSC, is a medical student at St George's, University of London, UK. **Rebecca Cox**, BSc, MSc, is a Clinical Research Trials Assistant at South West London and St George's Mental Health NHS Trust, Research & Development, UK. **Heloise Mongue-Din**, PharmD, PhD, is Research and Development Governance and Clinical Trials Manager at South West London and St George's Mental Health NHS Trust, UK. **Robert Lawrence** is Consultant and Honorary Senior Lecturer Psychiatry in Old Age and Neuropsychiatry, and Associate Medical Director for Research and Development at South West London and St George's Mental Health NHS Trust, UK
